# Tranexamic acid is associated with improved hemostasis in elderly patients undergoing coronary-artery surgeries in a retrospective cohort study

**DOI:** 10.3389/fsurg.2023.1117974

**Published:** 2023-02-21

**Authors:** Enshi Wang, Yang Wang, Yuan Li, Shengshou Hu, Su Yuan

**Affiliations:** ^1^ Department of Cardiovascular Surgery, Fuwai Hospital, Chinese Academy of Medical Sciences, Peking Union Medical College, Beijing, China; ^2^ Medical Research & Biometrics Center, National Center for Cardiovascular Diseases, Beijing, China; ^3^ Department of Anesthesiology, Fuwai Hospital, Chinese Academy of Medical Sciences, Peking Union Medical College, Beijing, China

**Keywords:** elderly patients, coronary artery bypass grafting, tranexamic acid, blood loss, blood transfusion, thromboembolic events, propensity score matching

## Abstract

**Background:**

More elderly patients undergo coronary artery bypass surgery (CABG) than younger patients. Whether tranexamic acid (TA) is still effective and safe in elderly patients undergoing CABG surgeries is still unclear.

**Methods:**

In this study, a cohort of 7,224 patients ≥70 years undergoing CABG surgery were included. Patients were categorized into the no TA group, TA group, high-dose group, and low-dose group according whether TA was administered and the dose administered. The primary endpoint was blood loss and blood transfusion after CABG. The secondary endpoints were thromboembolic events and in-hospital death.

**Results:**

The blood loss at 24 and 48 h and the total blood loss after surgery in patients in the TA group were 90, 90, and 190 ml less than those in the no-TA group, respectively (*p* < 0.0001). The total blood transfusion was reduced 0.38-fold with TA administration compared to that without TA (OR = 0.62, 95% CI 0.56–0.68, *p* < 0.0001). Blood component transfusion was also reduced. High-dose TA administration reduced the blood loss by 20 ml 24 h after surgery (*p* = 0.032) but had no relationship with the blood transfusion. TA increased the risk of perioperative myocardial infarction (PMI) by 1.62-fold [*p* = 0.003, OR = 1.62, 95% CI (1.18–2.22)] but reduced the hospital stay time in patients who were administered TA compared to that of patients who did not receive TA (*p* = 0.026).

**Conclusion:**

We revealed that elderly patients undergoing CABG surgeries had better hemostasis after TA administration but increased the risk of PMI. High-dose TA was effective and safe compared with low-dose TA administration in elderly patients undergoing CABG surgery.

## 1. Introduction

With economic and medical development, the aging of society will inevitably lead to an increasing proportion of patients over 70 years who undergo cardiac surgery (1). As the population ages, the incidence of age-related complications (including diabetes, peripheral vascular diseases, kidney diseases and cardiovascular diseases) has increased. The number of potential candidates for geriatric surgery is increasing, as approximately a quarter of people over the age of 75 develop cardiovascular disease, and more than half of all heart surgeries are performed in this age group (2). However, the long-term and short-term mortality and morbidity in elderly patients were only acceptable if accurate selection, a multifactorial risk evaluation, was adopted and nonelective operations were not performed (3). Compared to younger patients, elderly patients had a higher re-exploration rate in cardiac surgery (4, 5), which was associated with a higher blood transfusion rate. Based on the natural properties in older patients, bleeding and blood transfusion were increased during cardiac surgeries and thereby were combined with more complications, more deaths and greater costs (6, 7).

To avoid bleeding and blood comorbidities in the aged population, antifibrinolytic drugs, such as tranexamic acid (TA), have been used during cardiac surgery to maintain hemostasis (8). As a double-edged sword, TA also has a risk of thromboembolic complications after cardiac surgery (9). Nevertheless, controversy regarding TA thrombosis during cardiac surgery remains (9–13). In elderly patients, the physiological reserve decreases, and the reduction in coronary flow reserve and the progression of atherosclerosis lead to a higher coronary artery disease incidence (2). In addition, during coronary artery bypass grafting (CABG), the activation of the hemostatic system, which showed more obvious fibrinolysis and platelet activation, increased in elderly patients compared with young patients (14). Furthermore, TA more easily accumulated in older patients due to the higher renal dysfunction complications in the perioperative period, and 90% of TA was unchanged and eliminated by the patients’ kidneys (2, 15, 16). All the above factors pose a higher thromboembolic risk in elderly patients undergoing CABG with TA administration. Therefore, the blood management and safety issues of TA in the older population undergoing CABG were taken into consideration in this study.

## 2. Materials and method

### 2.1. Patient information

**Figure 1:**
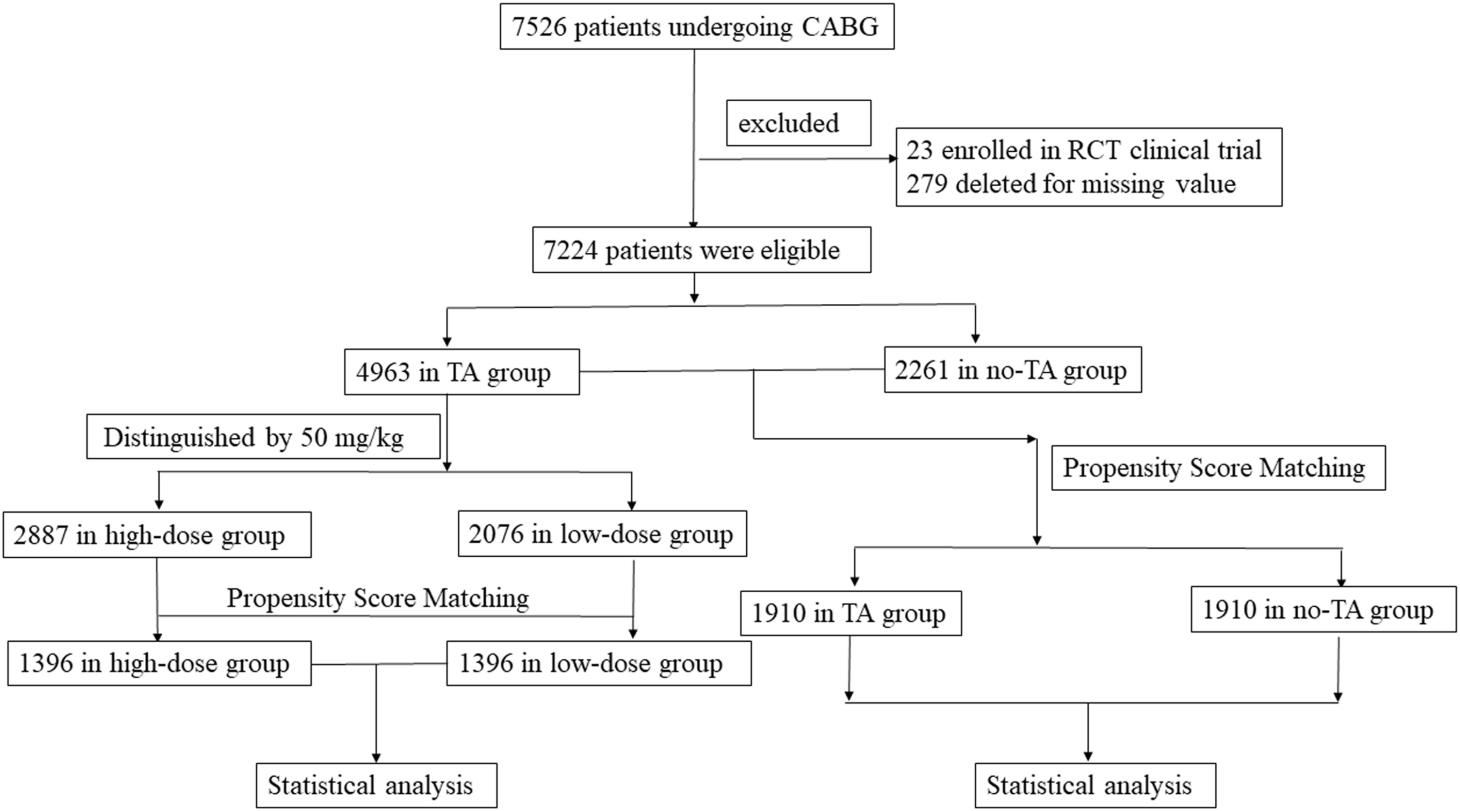
Flowchart of elderly patients undergoing CABG surgery with or without TA administration. This study included 7,526 patients older than 70 years treated by CABG surgery. Patients enrolled in RCT studies or with missing values were removed from this study. Finally, a total of 7,224 elderly patients were selected for this study. Among them, the TA group had 4,963 patients, and the no-TA group had 2,261 patients. In the TA group, 2,887 patients were administered a TA dose ≥50 mg/kg, and the remaining 2,076 patients were administered a TA dose <50 mg/kg. The outcomes between the TA and no-TA groups or the high-dose and low-dose groups were compared by PSM and subsequent statistical analysis. CABG, coronary artery bypass grafts; RCT, random control trial; TA, tranexamic acid; PSM, propensity score matching.

From January 2009 to December 2019, 7,526 patients ≥70 years undergoing CABG surgery were considered for inclusion in this study. Among them, 23 patients were excluded due to enrollment in RCT clinical trials, and 279 patients were excluded due to missing values. The remaining 7,224 patients (95.98%) were ultimately included in this study (Figure 1). According to the administration of TA, the elderly patients were divided into two groups. The TA group consisted of 4,963 patients, and the no-TA group included 2,261 patients. After propensity score matching (PSM), the TA group included 1,910 patients, and the non-TA group included 1,910 patients (Figure 1, Table 1). In the TA group, a total of 4,963 patients were divided into the high-dose group and the low-dose group with a cutoff value of 50 mg/kg according to previous studies (17–23). Patients who received a TA dose greater than 50 mg/kg were categorized as the high-dose TA group, and patients who were administered a TA dose less than 50 mg/kg were defined as the low-dose TA group. In this study, the high-dose TA group included 2,887 patients, and the low-dose TA group included 2,076 patients. After PSM, there were 1,396 patients in the high-dose and low-dose groups (Figure 1, Table 2). The study was carried out according to the guidelines of the Declaration of Helsinki 1964 and its subsequent amendments. The Medical Ethics Committee of Fuwai Hospital approved the protocol. The requirement for informed consent was waived by the Medical Ethics Committee due to the retrospective nature of the study.

**Table 1:** The baseline information of elderly patients undergoing CABG with or without TA administration.

Characteristics	Before matching			After matching		
	TA group (*n* = 4,963)	No TA group (*n* = 2,261)	*p* value	TA group (*n* = 1,910)	No TA group (*n* = 1,910)	*p* value
Age (year), mean ± SD	73.33 ± 2.93	73.49 ± 3.00	0.028	73.54 ± 3.00	73.49 ± 3.00	0.045
BMI (kg/m^2^), mean ± SD	25.00 ± 3.04	24.99 ± 3.03	0.916	24.95 ± 3.08	24.98 ± 3.06	0.941
Male sex, *n* (%)	3,415 (68.8)	1,620 (71.6)	0.015	1,364 (71.4)	1,341 (70.2)	0.433
NYHA III-V, *n* (%)	1,657 (33.4)	598 (26.4)	<0.0001	529 (27.7)	541 (28.3)	0.681
LV dysfunction (ejection fraction <40%), *n* (%)	175 (3.5)	60 (2.7)	0.053	56 (2.9)	55 (2.9)	1.000
**Preexisting medical conditions, *n* (%)**						
Insulin dependent diabetes	510 (10.3)	180 (8.0)	0.002	161 (8.4)	168 (8.8)	0.732
Hyperlipidemia	3,303 (66.6)	1,274 (56.3)	<0.0001	1,178 (61.7)	1,141 (59.7)	0.226
Hypertension	3,339 (67.3)	1,482 (65.5)	0.147	1,297 (67.9)	1,268 (66.4)	0.331
Chronic kidney disease	661 (13.3)	304 (13.4)	0.883	259 (13.6)	256 (13.4)	0.925
COPD	135 (2.7)	69 (3.1)	0.430	53 (2.8)	60 (3.1)	0.565
Peripheral vascular disease	775 (15.6)	270 (11.9)	<0.0001	258 (13.5)	249 (13.0)	0.699
Cerebrovascular accident	826 (16.6)	338 (14.9)	0.069	245 (18.1)	293 (15.3)	0.028
Previous cardiac surgery	242 (4.9)	67 (3.0)	<0.0001	63 (3.3)	66 (3.5)	0.855
Preoperative atrial fibrillation	286 (5.8)	81 (3.6)	<0.0001	77 (4.0)	73 (3.8)	0.803
Acute coronary syndrome	988 (19.9)	496 (21.9)	0.048	405 (21.2)	410 (21.5)	0.874
Left main stem disease	642 (12.9)	537 (23.8)	<0.0001	382 (20.0)	374 (19.6)	0.767
Three-vessel disease	3,763 (75.8)	1,711 (75.7)	0.893	1,452 (76.0)	1,441 (75.4)	0.703
Preoperative IABP	69 (1.4)	31 (1.4)	0.948	29 (1.5)	27 (1.4)	0.894
Time between CAG and operation less than 3 days	164 (3.3)	83 (3.7)	0.427	41 (2.1)	72 (3.8)	0.004
No. of risk factors for bleeding			0.010			0.816
0–1	1,418 (28.6)	656 (29.0)		543 (28.4)	542 (28.4)	
2–3	3,286 (66.2)	1,524 (67.4)		1,281 (67.1)	1,292 (67.6)	
4–5	259 (5.2)	81 (3.6)		86 (4.5)	76 (4.0)	
**Preoperative medications, *n* (%)**						
Aspirin within last 5 days	551 (11.1)	245 (10.8)	0.738	240 (12.6)	219 (11.5)	0.318
Clopidogrel within last 5 days	676 (13.6)	337 (14.9)	0.145	309 (16.2)	296 (15.5)	0.596
Ticagrelor within last 5 days	19 (0.4)	3 (0.1)	0.074	4 (0.2)	3 (0.2)	1.000
LWMH within 24 h	1,319 (26.6)	620 (27.4)	0.452	507 (26.5)	509 (26.6)	0.971
ACEI or ARB	1,921 (38.7)	1,067 (47.2)	<0.0001	854 (44.7)	865 (45.3)	0.741
Nitrate	4,583 (92.3)	2,152 (95.2)	<0.0001	1,808 (94.7)	1,808 (94.7)	1.000
Beta-blocker	4,035 (81.3)	1,909 (84.4)	0.001	1,589 (83.2)	1,594 (83.5)	0.863
Calcium-channel blocker	1,210 (24.4)	662 (29.3)	<0.0001	521 (27.3)	527 (27.6)	0.855
Statin	3,928 (79.1)	1,667 (73.7)	<0.0001	1,440 (75.4)	1,435 (75.1)	0.879
**Preoperative laboratory tests**						
eGFR (ml/min/1.73 m^2^), mean ± SD	83.17 ± 21.59	82.48 ± 21.25	0.210	83.34 ± 21.65	82.65 ± 21.21	0.028
Hb male/female < 130/120 g/L), *n* (%)	1,146 (23.1)	544 (24.1)	0.367	427 (22.4)	428 (22.4)	1.000
Thrombocytopenia, *n* (%)	738 (14.9)	333 (14.7)	0.875	303 (15.9)	274 (14.3)	0.210
Propensity score, mean ± SD	0.74 ± 0.16	0.62 ± 0.17	<0.0001	0.62 ± 0.17	0.62 ± 0.17	<0.0001

TA, tranexamic acid; SD, standard deviation; BMI, body mass index; NYHA, New York Heart Association (classification); LV, left ventricle; COPD, chronic obstructive pulmonary disease; IABP, intra-aortic balloon pump; CAG, coronary angiography; LMWH, low-molecular-weight heparin; ACEI, angiotensin converting enzyme inhibitor; ARB, angiotensin-receptor blocker; eGFR, estimated Glomerular filtration rate; Hb, hemoglobin.

**Table 2:** Characteristics of elderly patients in the high-dose and low-dose TA group.

Characteristics	Before matching			After matching		
	High-dose (*n* = 2,887)	Low-dose (*n* = 2,076)	*p* value	High-dose (*n* = 1,396)	Low-dose (*n* = 1,396)	*p* value
Age (year), mean ± SD	73.32 ± 2.96	73.35 ± 2.90	<0.0001	73.35 ± 3.01	73.33 ± 2.90	0.827
BMI (kg/m^2^), mean ± SD	24.38 ± 3.06	25.86 ± 2.79	<0.0001	25.26 ± 3.02	25.26 ± 2.58	0.937
Male sex, *n* (%)	1,061 (36.8)	487 (23.5)	<0.0001	384 (27.5)	385 (27.6)	1.000
NYHA III-V, *n* (%)	1,079 (37.4)	578 (27.8)	<0.0001	463 (33.2)	418 (29.9)	0.075
LV dysfunction (ejection fraction <40%), *n* (%)	106 (3.7)	69 (3.3)	0.512	47 (3.4)	51 (3.7)	0.757
**Preexisting medical conditions, *n* (%)**						
Insulin dependent diabetes	337 (11.7)	173 (8.3)	<0.0001	151 (10.8)	138 (9.9)	0.449
Hyperlipidemia	1,970 (68.2)	1,333 (64.2)	0.003	946 (67.8)	941 (67.4)	0.872
Hypertension	1,927 (66.7)	1,412 (68.0)	0.348	969 (69.4)	952 (68.2)	0.519
Chronic kidney disease	396 (13.7)	265 (12.8)	0.330	183 (13.1)	163 (11.7)	0.282
COPD	72 (2.5)	63 (3.0)	0.248	37 (2.7)	48 (3.4)	0.272
Peripheral vascular disease	435 (15.1)	340 (16.4)	0.210	215 (15.4)	217 (15.5)	0.958
Cerebrovascular accident	468 (16.2)	358 (17.2)	0.335	240 (17.2)	232 (16.6)	0.724
Previous cardiac surgery	155 (5.4)	87 (4.2)	0.057	62 (4.4)	66 (4.7)	0.784
Preoperative atrial fibrillation	204 (7.1)	82 (3.9)	<0.0001	61 (4.4)	62 (4.4)	1.000
Acute coronary syndrome	540 (18.7)	448 (21.6)	0.012	280 (20.1)	287 (20.6)	0.776
Left main stem disease	338 (11.7)	304 (14.6)	0.002	193 (13.8)	201 (14.4)	0.703
Three-vessel disease	2,194 (76.0)	1,569 (75.6)	0.735	1,080 (77.4)	1,073 (76.9)	0.786
Preoperative IABP	42 (1.5)	27 (1.3)	0.647	22 (1.6)	15 (1.1)	0.324
Time between CAG and operation less than 3 days	102 (3.5)	62 (3.0)	0.288	47 (3.4)	46 (3.3)	1.000
No. of risk factors for bleeding			<0.0001			1.000
0–1	772 (26.7)	646 (31.1)		421 (30.2)	421 (30.2)	
2–3	1,939 (67.2)	1,347 (64.9)		909 (65.1)	912 (65.3)	
4–5	176 (6.1)	83 (4.0)		66 (4.7)	63 (4.5)	
**Preoperative medications, *n* (%)**						
Aspirin within last 5 days	298 (10.3)	253 (12.2)	0.039	173 (12.4)	163 (11.7)	0.597
Clopidogrel within last 5 days	355 (12.3)	321 (15.5)	0.001	208 (14.9)	204 (14.6)	0.870
Ticagrelor within last 5 days	11 (0.4)	8 (0.4)	0.981	4 (0.3)	7 (0.5)	0.549
LWMH within 24 h	795 (27.5)	524 (25.2)	0.071	366 (26.2)	370 (26.5)	0.899
ACEI or ARB	1,050 (36.4)	871 (42.0)	<0.0001	570 (40.8)	567 (40.6)	0.939
Nitrate	2,620 (90.8)	1,963 (94.6)	<0.0001	1,316 (94.3)	1,315 (94.2)	1.000
Beta-blocker	2,318 (80.3)	1,717 (82.7)	0.031	1,145 (82.0)	1,153 (82.6)	0.722
Calcium-channel blocker	685 (23.7)	525 (25.3)	0.206	332 (23.8)	335 (24.0)	0.930
Statin	2,315 (80.2)	1,613 (77.7)	0.033	1,124 (80.5)	1,116 (79.9)	0.737
**Preoperative laboratory tests**						
eGFR (ml/min/1.73 m^2^), mean ± SD	83.38 ± 21.79	82.87 ± 21.29	0.408	83.76 ± 21.58	83.39 ± 21.27	0.223
Hb male/female < 130/120 g/L), *n* (%)	681 (23.6)	465 (22.4)	0.327	341 (24.4)	311 (22.3)	0.195
Thrombocytopenia, *n* (%)	410 (14.2)	328 (15.8)	0.119	198 (14.2)	203 (14.5)	0.825
Propensity score, mean ± SD	0.68 ± 0.20	0.54 ± 0.19	<0.0001	0.55 ± 0.19	0.54 ± 0.19	<0.0001

TA, tranexamic acid; SD, standard deviation; BMI, body mass index; NYHA, New York Heart Association (classification); LV, left ventricle; COPD, chronic obstructive pulmonary disease; IABP, intra-aortic balloon pump; CAG, coronary angiography; LMWH, low-molecular-weight heparin; ACEI, angiotensin converting enzyme inhibitor; ARB, angiotensin-receptor blocker; eGFR, estimated Glomerular filtration rate; Hb, hemoglobin.

### 2.2. Operation details

Colleagues collected all patients’ data from the electronic hospital records in the information center of our hospital. All patients in this study underwent CABG surgeries. Among them, isolated CABG accounted for 85.8% of patients in the TA group and 92.2% of patients in the no-TA group (Supplementary Table S1). The remaining CABG procedures were carried out with aneurysm surgeries, valve surgery, or aortic or arch surgery. Elective surgery accounted for 97.0% of surgeries in the TA group and 95.4% of surgeries in the no-TA group (Supplementary Table S1). On-pump surgeries were performed on 58.5% of patients in the TA group and 36.2% of patients in the no-TA group (Supplementary Table S1). Isolated CABGs accounted for 81.8% of patients in the high-dose TA group and 97.2% of patients in the low-dose TA group. In the high-dose TA group, 65.5% of surgeries were on-pump surgeries, while in the low-dose TA group, 48.8% of surgeries were on-pump surgeries (Supplementary Table S2).

We defined high-risk surgery as emergent CABG surgery, CABG with a history of previous cardiac surgery, CABG plus aortic or arch operation, or CABG plus valve surgery. Open chamber surgeries included CABG plus aneurysm resection, aortic operation or valve surgery. Perioperative myocardial infarction (PMI) is diagnosed by an isolated elevation of creatine kinase myocardial isoenzyme (CK-MB) to ≥10 × 99th percentile upper reference limit (URL) or cardiac troponin (cTn) (I or T) to ≥70 × URL during the first 48 h following CABG surgery with or without ECG or imaging changes of MI (24, 25).

### 2.3. Hemostasis during surgery

TA was administered after anesthesia induction. The administration of TA was under the consideration of anesthesiologists. TA was given at a dose of 54.83 mg/kg (median) with an interquartile range (IQR) from 42.73 to 72.46 mg/kg in elderly patients. The median TA dose was 70.42 mg/kg (IQR 60.24–80.64 mg/kg) in patients in the high-dose group, while the median was 40.76 mg/kg (IQR 35.30–44.78 mg/kg) in patients in the low-dose group. Cell salvage was used during the operation for blood conservation (Fresenius Kabi C.A.T.S.®^plus^, Fresenius Kabi AG, Bad Homburg, Germany). For on-pump surgeries, the activated clotting time (ACT) was maintained higher than 410 s with a heparin dose of 400 IU/kg, and for off-pump surgeries, the ACT was higher than 300 s with a heparin dose of 200 IU/kg. Additional doses of heparin were given according to the dynamic changes in act during the operation. The ratio of protamine to heparin (1 mg protamine: 100 units heparin) was 1:1; this ratio was used for neutralization. More protamine was added in consideration of hemostasis, ACT value and the recommendation of surgeons.

### 2.4. Outcome definition

The primary outcome was defined as chest tube drainage and blood transfusion after surgery. The chest tube drainage volume at 24 and 48 h and the total chest tube drainage after CABG were considered as the blood loss after surgery. Blood transfusion after CABG included red blood cell (RBC) infusion, fresh frozen plasma (FFP) infusion and platelet (PLT) infusion. Secondary outcomes were safety issues, including in-hospital deaths and thromboembolic events [perioperative myocardial infarction (PMI), stroke, acute renal injury (AKI), and pulmonary embolism]. The details about the definitions are provided in the supplementary materials.

### 2.5. Statistical analysis

The normal distribution was expressed as the means ± standard deviations (SDs), and nonnormal distributions were expressed as the medians and interquartile ranges (IQRs). Categorical variables are presented as numbers and percentages.

Before PSM, the baseline information between the TA group and no-TA group or the high-dose TA and low-dose TA groups was compared by Student’s *t* test for normally distributed continuous variables, the Mann‒Whitney *U* test for nonnormally distributed variables, and the *χ*^2^ test or Fisher’s exact test for categorical variables. A paired *t* test was used for normally distributed data, the Wilcoxon rank test was used for nonnormally distributed data, and McNemar’s test was used for categorical data after PSM.

Conditional logistic regression was performed in the binary outcome evaluation with an odds ratio (OR) and 95% confidence intervals (CIs) after PSM.

**Figure 2:**
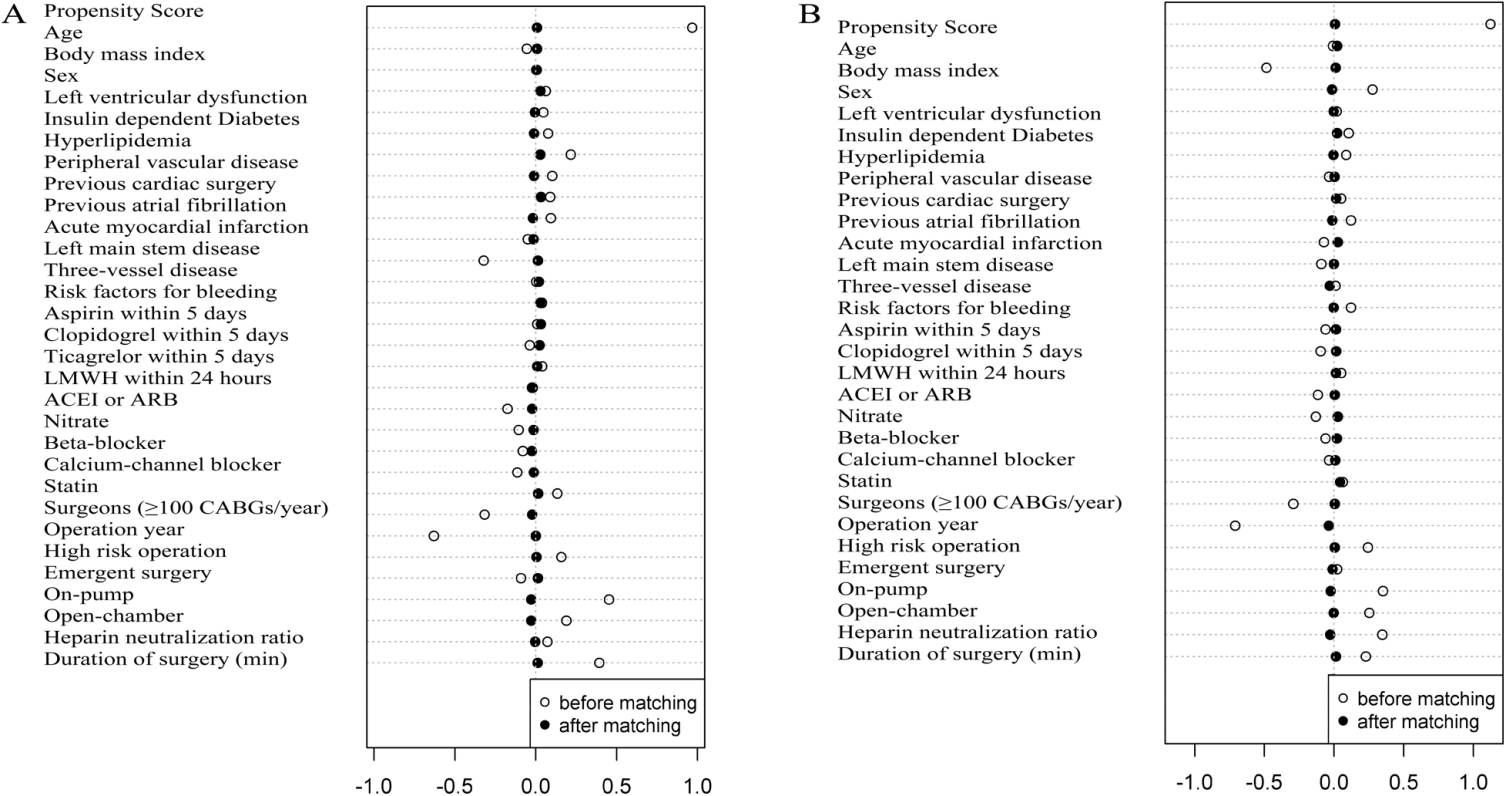
Elderly patients undergoing CABG surgery with or without TA or with high- or low-dose TA administration were matched by propensity score. (**A**) PSM was used and well balanced in elderly patients with or without TA administration. The standardized difference was less than 0.1 in 30 variables; (**B**) elderly patients with high-dose and low-dose TA administration were matched well by propensity score. The standardized difference was less than 0.1 in all 29 covariates. Insulin-dependent diabetes was defined as the treatment of patients’ diabetes dependent on insulin therapy. Left ventricular dysfunction was defined as a patient’s ejection fraction ≤40%. Surgeons who performed more than 100 CABG surgeries per year assigned a value of “1”, and surgeons who performed less than 100 CABG surgeries per year were assigned a value of “0”. The risk factors for bleeding were age older than 70 years, female sex, low-molecular-weight heparin or an antiplatelet drug less than 5 days before surgery, renal impairment (estimated glomerular filtration rate, <60 ml per minute), and insulin-dependent diabetes.

Propensity score matching was performed according to 1:1 matching between the TA and no-TA groups or the high-dose and low-dose groups. The caliper width was 0.01, and the nearest-neighbor matching method without replacement was selected. There were 30 variables chosen based on the clinical and statistical significance in the PSM of the TA and no-TA groups (Supplementary Table S3), while 29 variables were matched in the high-dose and low-dose groups (except ticagrelor within 5 days) (Supplementary Table S4). A total of 1,910 patients in the TA group and 1,910 patients in the no-TA group were matched well. Balances were well maintained in the demographical and perioperative data with standardized differences <0.1 (Figure 2A). Additionally, 1,396 patients in the high-dose group and 1,396 in the low-dose group were matched and balanced well with standardized differences <0.1 (Figure 2B).

Binary logistic regression was used in the sensitivity analysis in the entire elderly cohort. Covariates were 30 variables and TA administration. Binary logistic regression was also applied to patients in the TA cohort with 29 variables and TA dosage as covariates. The dependent variables were binary outcomes. The “enter” method was used, and adjusted ORs and 95% CIs of outcome variables were generated.

*p* < 0.05 was considered statistically significant. All statistical analyses were carried out with IBM SPSS Statistics for Windows, version 22.0 (IBM Corp., Armonk, NY, USA).

## 3. Results

### 3.1. The primary outcome in the TA and no-TA groups

After PSM, TA application was not related to the re-exploration rate due to massive hemorrhage and pericardial tamponade (*p* = 0.663) (Table 3). However, the blood loss after surgery was significantly reduced in the TA groups compared to that in the no-TA group (Table 3). The blood loss in 24 h after surgery in the TA group was 90 ml less than that in the no-TA group (*p* < 0.0001). The blood loss in 48 h after surgery in patients with TA was also 90 ml less than that in those without TA (*p* < 0.0001). After surgery, the total blood loss for patients with TA was 190 ml less than that in those without TA (*p* < 0.0001).

**Table 3:** Adjusted odds ratios in elderly patients for primary and secondary endpoints between the TA group and no TA group by PSM and logistic regression.

Outcomes	TA group (*n* = 1,910)	No TA group (*n* = 1,910)	OR (95% CI) by PSM	*p* value	OR (95% CI) by logistic regression	*p* value
**Primary outcome**						
**Blood loss after operation**						
Reoperation due to major hemorrhage or cardiac tamponade, *n* (%)	40 (2.1)	44 (2.3)	0.91 (0.59–1.40)	0.663	0.93 (0.64–1.35)	0.705
Blood loss in 24 h after surgery (ml), mean ± SD	450 (320–600)	530 (470–750)		<0.0001		
Blood loss in 48 h after surgery (ml), mean ± SD	720 (530–940)	810 (710–1,072)		<0.0001		
Total Blood loss after surgery (ml), mean ± SD	940 (680–1,340)	1,130 (900–1,430)		<0.0001		
**Blood transfusion after operation, *n* (%)**						
Blood transfusion	658 (34.5)	1,063 (55.7)	0.62 (0.56–0.68)	<0.0001	0.38 (0.34–0.43)	<0.0001
RBC	568 (29.7)	879 (46.0)	0.65 (0.58–0.72)	<0.0001	0.44 (0.39–0.50)	<0.0001
FFP	296 (15.5)	614 (32.1)	0.48 (0.42–0.55)	<0.0001	0.37 (0.32–0.42)	<0.0001
PLT	63 (3.3)	78 (4.1)	0.81 (0.58–1.13)	0.207	0.75 (0.54–1.04)	0.081
**Secondary outcome, *n* (%)**	235 (12.3)	212 (11.1)	1.11 (0.92–1.34)	0.277	1.08 (0.89–1.27)	0.475
Hospital death	10 (0.5)	14 (0.7)	0.71 (0.32–1.61)	0.416	0.59 (0.27–1.30)	0.191
Myocardial infarction	102 (5.3)	63 (3.3)	1.62 (1.18–2.22)	0.003	1.45 (1.08–1.95)	0.014
Stroke	25 (1.3)	22 (1.2)	1.14 (0.64–2.02)	0.662	0.97 (0.59–1.59)	0.905
Acute renal injury	130 (6.8)	130 (6.8)	1.00 (0.78–1.28)	1.000	1.02 (0.81–1.28)	0.864
Pulmonary embolism	4 (0.2)	3 (0.2)	1.33 (0.30–5.96)	0.706	1.06 (0.30–3.70)	0.930
**Postoperative course**						
Intensive care (h), median (IQR)	48 (24–96)	48 (24–96)		0.004		
Hospital stay (day), mean ± SD	17.50 ± 8.50	18.13 ± 9.25		0.026		
**Adverse events after surgery, *n* (%)**						
Death from any cause within 30 days	25 (1.3)	24 (1.3)	1.04 (0.60–1.82)	0.886	0.82 (0.48–1.41)	0.474
Seizure	1 (0.1)	4 (0.2)	0.25 (0.03–2.24)	0.215	0.44 (0.10–1.87)	0.256

TA, tranexamic acid; RBC, red blood cell; FFP, fresh frozen plasma; PLT, platelet; OR, odds ratio; CI, confidence interval; IQR, interquartile range; SD, standard deviation.

In accordance with the decline in blood loss after TA administration, blood transfusion after surgery was also reduced in the TA group compared to that in the no-TA group (Table 3). The total blood transfusion was reduced 0.38-fold with TA administration compared to that in those without TA (OR = 0.62, 95% CI 0.56–0.68, *p* < 0.0001). Blood component transfusion was also reduced. TA administration reduced the RBC transfusion rate 0.35-fold (OR = 0.65, 95% CI 0.58–0.72, *p* < 0.0001); the FFP transfusion rate was reduced 0.52-fold (OR = 0.48, 95% CI 0.42–0.55, *p* < 0.0001) by TA administration, but there was no reduction in the PLT transfusion rate after using TA (OR = 0.81, 95% CI 0.58–1.13, *p* = 0.207).

The sensitivity analysis yielded results similar to those of the PSM analysis (Table 3).

### 3.2. The primary outcome in the high-dose and low-dose TA groups

After PSM, the reoperation rate in the high-dose TA group was not different from that in the low-dose group [*p* = 0.397, OR = 1.27, 95% CI (0.73–2.23)] (Table 4). However, the 24-h blood loss after the operation in the high-dose TA group was 20 ml less than that in the low-dose TA group statistically (*p* = 0.032) (Table 4). The blood loss within 48 h in the high-dose group was also lower than that in the low-dose group statistically (median 690 vs. 730 ml, *p* = 0.014). There were no differences in the total blood loss between the two groups (*p* = 0.174). The blood transfusion rate or components were not associated with the TA dosage (*p* > 0.05).

**Table 4:** Adjust odds ratios in elderly patients for primary and secondary endpoints between the high-dose and low-dose TA subgroups by PSM.

Outcome	TA group (*n* = 1,396)	No-TA group (*n* = 1,396)	OR (95% CI) by PSM	*p* value	OR (95% CI) by logistic regression	*p* value
**Primary outcome**						
**Blood loss after operation**						
Reoperation due to major hemorrhage or cardiac tamponade, *n* (%)	28 (2.0)	22 (1.6)	1.27 (0.73–2.23)	0.397	1.20 (0.75–1.91)	0.448
Blood loss in 24 h after surgery (ml), mean ± SD	430 (310–590)	450 (320–620)		0.032		
Blood loss in 48 h after surgery (ml), mean ± SD	690 (520–900)	730 (530–957)		0.014		
Total Blood loss after surgery (ml), mean ± SD	930 (670–958)	960 (686–1,364)		0.174		
**Blood transfusion after operation, *n* (%)**						
Blood transfusion	479 (34.3)	477 (34.2)	1.00 (0.89–1.14)	0.948	1.04 (0.90–1.20)	0.605
RBC	430 (30.8)	414 (29.7)	1.04 (0.91–1.19)	0.582	1.10 (0.95–1.28)	0.206
FFP	202 (14.5)	226 (16.2)	0.89 (0.74–1.08)	0.246	0.92 (0.77–1.11)	0.384
PLT	39 (2.8)	42 (3.0)	0.93 (0.60–1.44)	0.739	1.06 (0.71–1.60)	0.770
**Secondary outcome, *n* (%)**	170 (12.2)	169 (12.1)	1.01 (0.81–1.25)	0.957	1.02 (0.84–1.25)	0.832
Hospital death	6 (0.4)	9 (0.6)	0.67 (0.24–1.87)	0.442	0.50 (0.17–1.48)	0.207
Myocardial infarction	74 (5.3)	71 (5.1)	1.04 (0.75–1.44)	0.803	1.10 (0.83–1.46)	0.520
Stroke	20 (1.4)	15 (1.1)	1.33 (0.68–2.60)	0.400	1.26 (0.69–2.29)	0.450
Acute renal injury	86 (6.2)	99 (7.1)	0.87 (0.65–1.16)	0.340	0.93 (0.71–1.22)	0.607
Pulmonary embolism	6 (0.4)	3 (0.2)	2.00 (0.50–8.00)	0.327	1.24 (0.29–5.35)	0.773
**Postoperative course**						
Intensive care (h), median (IQR)	48 (24–96)	48 (24–96)		0.201		
Hospital stay (day), mean ± SD	17.38 ± 8.27	16.76 ± 7.67		0.041		
**Adverse events after surgery, *n* (%)**						
Death from any cause within 30 days	15 (1.1)	17 (1.2)	0.88 (0.44–1.77)	0.724	0.67 (0.36–1.26)	0.215
Seizure	2 (0.1)	0 (0.0)	-	0.500	0.92 (0.11–7.92)	0.938

TA, tranexamic acid; RBC, red blood cell; FFP, fresh frozen plasma; PLT, platelet; OR, odds ratio; CI, confidence interval; IQR, interquartile range; SD, standard deviation.

### 3.3. Safety issues in the TA and no-TA groups

The secondary outcome was not associated with TA administration [*p* = 0.277, OR = 1.11, 95% CI (0.92–1.34)] (Table 3). No differences were noted between the two groups for stroke, AKI, pulmonary embolism or in-hospital death. However, TA increased the risk of PMI 1.62-fold [*p* = 0.003, OR = 1.62, 95% CI (1.18–2.22)] (Table 3).

The administration of TA also reduced the hospital stay length compared to that of patients without TA (*p* = 0.026). TA was not associated with the risk of death within 30 days or seizure (*p* > 0.05).

The results from the sensitivity analysis were similar to the PSM results (Table 3).

### 3.4. Safety issues in the TA dosage group

The TA dosage did not influence the secondary outcomes or its constitutive components (*p* > 0.05) (Table 4). No differences were observed in death within 30 days or seizure risk between the high- and low-dose groups (*p* > 0.05). However, high-dose TA administration reduced the length of hospital stay compared to the low-dose subsets (16.76 ± 7.67 vs. 17.38 ± 8.27 days, *p* = 0.041).

Similar results were obtained by sensitivity analysis (Table 4).

## 4. Discussion

This study evaluated the TA and dosage effects on hemostasis and safety issues in elderly patients undergoing CABG surgeries in a retrospective cohort study. TA decreased chest tube drainage and blood infusion in elderly patients. However, the PMI risk in patients who received TA was increased significantly. High-dose TA administration also reduced blood loss but was not associated with the risk of blood transfusion. The dosage effects had no associations with the risk of safety issues.

Elderly patients undergoing cardiac surgeries had a higher risk of re-exploration, thereby increasing the risk of blood transfusion (4, 5). In a meta-analysis of 557,923 patients undergoing cardiac surgery (26), the patients who underwent re-exploration were significantly older, and re-exploration significantly increased the risk of postoperative mortality and morbidity. Murphy et al. (7) revealed that RBC infusion in cardiac operations was closely related to infection and the incidence of postoperative ischemia, length of hospital stay, and increased early and late mortality and hospitalization expenses. Ibrahim et al. (27) also found in a 14,281-patient cohort who received cardiac surgery that RBC infusion became an independent risk factor for readmission and mortality. Elderly patients undergoing CABG manifested higher hemostatic activation than young patients for increased fibrinolysis and platelet activation (14). This situation undoubtedly leads to a higher blood transfusion risk. Guri et al. reported that the number of RBC transfusions was reduced by TA in 64 patients aged ≥70 years undergoing CABG plus aortic valve replacement (8). However, the small number of patients limits the clinical significance. In this study, a larger CABG cohort including 7,224 patients ≥70 years old revealed that TA administration significantly reduced the blood loss and transfusion rate compared that observed when TA was not administered. Therefore, TA fosters sound blood management effects in elderly patients and thereby could benefit the prognosis of those undergoing CABG surgeries.

The double-blade effects of TA were investigated since it was introduced into clinical application. There was no doubt that TA could maintain hemostasis during CABG surgeries in the studies conducted previously (9–11) and in this study. The evidence on the safety issues of TA during cardiac surgery did not lead to a consensus (9–13). Myers et al. (11) reported no associations between thromboembolic events and TA administration. However, our previous study found that the risk of PMI was significantly increased after TA administration (9). Furthermore, Zhou et al. found that intraoperative TA was associated with postoperative stroke in patients undergoing cardiac surgery. For elderly patients undergoing CABG surgery, less research on the safety issues of TA has been conducted. Elderly patients undergoing cardiac surgery had higher morbidity and mortality, and 78% of deaths and major complications occurred in patients ≥75 years (2). Wilson et al. found that patients undergoing CABG aged over 75 years had a longer hospital stay times and higher perioperative mortality than those under 75 years (28). Based on the pathophysiology of elderly patients undergoing CABG surgery and the TA properties of thrombosis, the emphasis was on the safety issues after TA administration in this subset.

Our study found that the TA group showed an increased risk of PMI during hospitalization. This result was different from the study by Myles et al. (11). The ATACAS trial (11) revealed that TA did not increase the risk of thrombotic complications within 30 days after CABG. We tried to explain it in three aspects. First, the CABG composition was different in the ATACAS trial from that in our retrospective data. The CABG in the ATACAS trial were mainly on-pump (97% in the TXA group vs. 96.8% in the placebo group). However, the on-pump rate in CABG surgeries was not so high in the real world. Our study found that after PSM, the on-pump CABG in TA group and no-TA group accounted for 38.7% and 40.2% respectively (Supplementary Table S1). Second, different definitions of PMI were used. The PMI in the ATACAS trial was defined a bit complicatedly. It included the dynamic change of creatine kinase isoenzyme (CK-MB) or Troponin I (cTnI), the ECG change, or autopsy results, and Troponin I > 10 ng/ml, or Troponin T > 4.0, or CKMB > three times upper reference limit (URL) at any time >12 h post-CABG. However, the derivation of ECG or imaging data was difficult to find in our large retrospective cohort spanning 11 years. So the definition in this clinical study was according to the Society for Cardiovascular Angiography and Interventions (SCAI), which is mainly based on cTnI ≥ 70 URL or CK-MB ≥ 10 URL in the 48-h post-operative period (24, 25). Third, different populations were focused on. In the study by Myles et al., the mean age in the TA group was 66.8 ± 9.8 years, while 67.0 ± 9.6 years in the placebo group. Our study focused on older patients ≥70 years old. So the mean age in the TA group was 73.54 ± 3.00 years, and 73.49 ± 3.00 in the no-TA group after PSM. Our study population was significantly older than those in the ATACAS trial. According to the three main differences from the ATACAS trial, we believed the inconsistency of PMI results between ATACAS trial and our study was reasonable.

The blood management guidelines have already led to widespread administration of TA in adult cardiac surgery (29), but no consensus has been reached on the TA administration scheme. Some clinical trials recommended a high dose of TA for effective blood management (30, 31). Sigaut et al. (30). found that, compared with a low dose (10 mg/kg bolus + 1 mg/kg/h infusion), a high dose of TA (30 mg/kg bolus + 16 mg/kg/h infusion) decreased blood transfusion needs, blood loss, and the reoperation rate. However, other researchers discovered that low-dose TA administration is sufficient to reduce postoperative blood loss and RBC infusion in cardiac surgery (32–34). A model-based meta-analysis comprised 49,817 patients undergoing cardiopulmonary bypass operations and discovered that low-dose TA administration could reduce bleeding outcomes without increasing seizure risk (32). Waldow et al. found that high-dose TA administration over 100 mg/kg was an independent risk predictor of early seizure, and the postoperative blood loss and blood infusion needs were similar between the low-dose and high-dose TA groups (33). The TA dosage effects in elderly patients have been less evaluated. In this study, we discovered that high-dose TA administration could significantly decrease blood loss, but the blood transfusion between the two dose groups was not different. For safety issues, TA dose was not associated with thromboembolic events, in-hospital death, seizure, ICU stay time, or death within 30 days; conversely, high-dose TA administration could reduce the hospital stay time. In this respect, high-dose TA administration ≥50 mg/kg was both effective and safe for elderly patients.

However, there were several limitations in this study. First, clinical studies and evidence on TA in cardiac surgery have increased over time. Even at present, the safety issue of TA application in cardiac surgery is still under debate (9, 13). This study ranged in duration from 2009 to 2019. During this time, many anesthesiologists participated in CABG surgeries, making it hard to explain why TA was used or not during CABG. Therefore, we would like to conclude that TA was used under the consideration of anesthesiologists’ judgment and preference. Second, the 11-year period inevitably makes it difficult to obtain all the variables we needed for this analysis. Third, although PSM was chosen to simulate an RCT, potential cofounding covariates were possibly present. Therefore, a large RCT is needed for further study. Fourth, the study population was from China. The Asians appeared to have the lowest BMI, whereas the African American and Hispanic adults have the highest (35). The mean BMI in this study was around 25 kg/m^2^ either in the TA group or the no-TA group. As we know, lower BMI leads to more bleeding after cardiac surgery even in elderly patients (36–38). Thereby, the discrepancy in BMI among different ethnic groups may lead to different blood management effects after TA usage. Hence we could not simply extend our results to the world without consideration of racial differences.

We revealed that elderly patients undergoing CABG surgery had better hemostasis after TA administration but had increased PMI risk. High-dose TA administration was effective and safe for elderly patients who underwent CABG surgery.

## Data Availability

The original contributions presented in the study are included in the article/**Supplementary Material**, further inquiries can be directed to the corresponding authors.
